# A comprehensive ensemble model for comparing the allosteric effect of ordered and disordered proteins

**DOI:** 10.1371/journal.pcbi.1006393

**Published:** 2018-12-03

**Authors:** Luhao Zhang, Maodong Li, Zhirong Liu

**Affiliations:** 1 College of Chemistry and Molecular Engineering, Peking University, Beijing, China; 2 Department of Chemistry, Princeton University, Princeton, NJ, United States of America; 3 Center for Quantitative Biology, Peking University, Beijing, China; 4 State Key Laboratory for Structural Chemistry of Unstable and Stable Species, Beijing National Laboratory for Molecular Sciences (BNLMS), Peking University, Beijing, China; Weill Medical College of Cornell University, UNITED STATES

## Abstract

Intrinsically disordered proteins/regions (IDPs/IDRs) are prevalent in allosteric regulation. It was previously thought that intrinsic disorder is favorable for maximizing the allosteric coupling. Here, we propose a comprehensive ensemble model to compare the roles of both order-order transition and disorder-order transition in allosteric effect. It is revealed that the MWC pathway (order-order transition) has a higher probability than the EAM pathway (disorder-order transition) in allostery, suggesting a complicated role of IDPs/IDRs in regulatory proteins. In addition, an analytic formula for the maximal allosteric coupling response is obtained, which shows that too stable or too unstable state is unfavorable to endow allostery, and is thus helpful for rational design of allosteric drugs.

## Author summary

Allosteric effect is an important regulation mechanism in biological processes, where the binding of a ligand at one site of a protein influences the function of a distant site. Conventionally, allostery was thought to originate from structural transition. However, in recent years, intrinsically disordered proteins (IDPs) were found to be widely involved in allosteric regulation in despite of their lack of ordered structure under physiological condition. It is still a mystery why IDPs are prevalent in allosteric proteins and how they differ from ordered proteins in allostery. Here, we propose a comprehensive ensemble model which includes both ordered and disordered states of a two-domain protein, and investigate the role of various state combinations in allosteric effect. By sampling the parameter space, we conclude that disordered proteins are less competitive than ordered proteins in performing allostery from a thermodynamic point of view. The prevalence of IDPs in allosteric regulation is likely determined by all their advantage, but not only by their capacity in endowing allostery.

## Introduction

**Fig 1:**
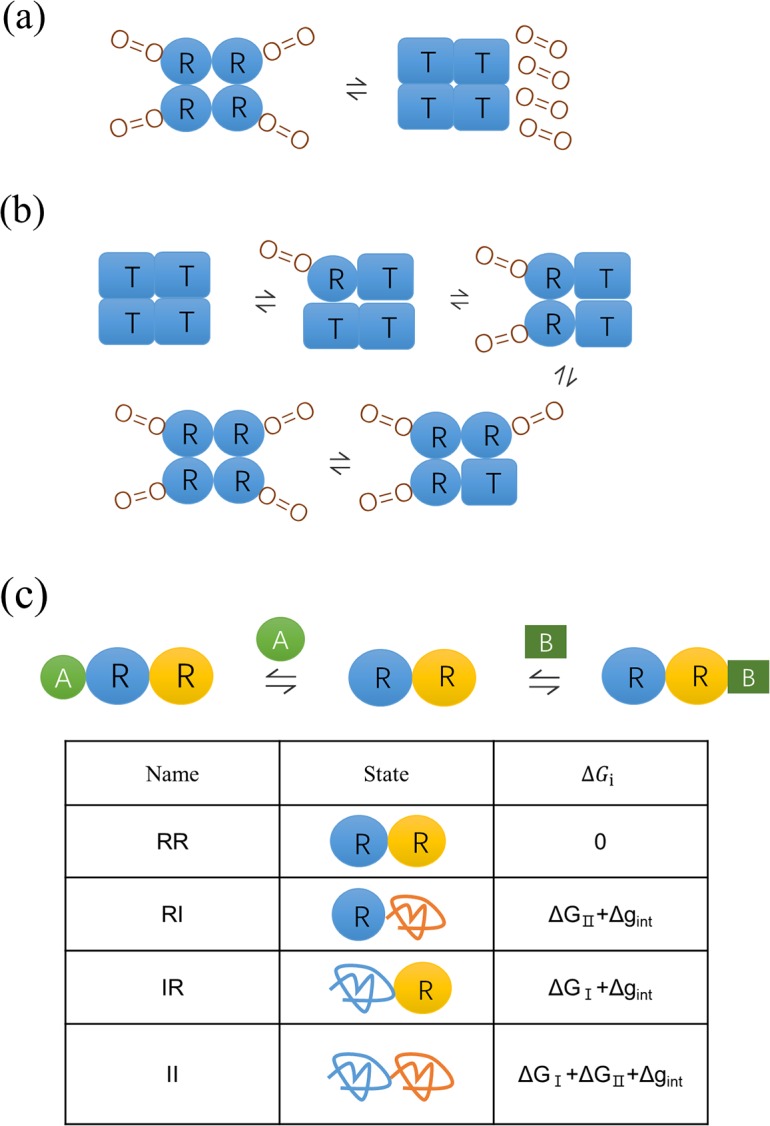
Schematic diagram of MWC, KNF and EAM models. (a) The MWC model for hemoglobin [3]. All four subunits are simultaneously in either an R (Relaxed) state (with a higher affinity for O_2_) or a T (Tense) state (with a lower affinity for O_2_). The cooperative effect happens when one O_2_ binds to the all-R state, shifts the chemical balance from all-T to all-R, then creates more favorable binding sites for subsequent O_2_. (b) The KNF model for hemoglobin [4]. It allows several intermediate states keeping balanced by several chemical equilibrium constants. (c) The EAM model for a two-domain protein [12]. Each domain can be in either an R (Relaxed) state or an I (Disordered) state, resulting in four possible combinations for protein states: RR, RI, IR and II. The corresponding probability *P*_*i*_ of four states is related to their free energy Δ*G*_*i*_ (relative to the RR state) as $P_{i}=e^{-\Delta G_{i}/RT}/Q$ where *Q* is the sum of statistical weights as $Q=\sum_{i}e^{-\Delta G_{i}/RT}$. The free energy Δ*G*_*i*_ were listed in the graphics, where Δ*G*_I_ and Δ*G*_II_ are the free energy of unfolding the R state of each domain, and Δ*g*_int_ is the free energy of breaking the interface interactions of ordered complex (RR). One domain (blue) in the R state can bind the allosteric ligand (A) while the other domain (yellow) in the R state can bind the substrate (B). The I state of each domain has no affinity to ligand and substrate.

Allosteric regulation is intrinsic to the control of many metabolic and signal-transduction pathways [1]. It is described as the effect that the binding of a ligand at one site of a protein influences the function of a distant site which binds with substrate [2]. In history, several models have been proposed illuminating possible mechanism of allostery. The classical MWC (Monod-Wyman-Changeux) [3] model explained the allosteric effect based on a cooperative conformational transition of protein oligomers. Taking hemoglobin binding with oxygen as an example (Fig 1(A)), the MWC model assumes that four subunits of hemoglobin are simultaneously in either a relaxed state (R state) or a tense state (T state), and oxygens bind preferentially to the R state which shifts the R-T equilibrium. With such a simple assumption, the MWC model nicely explained how the binding of oxygen at one site promotes the binding at a remote site. Later, the KNF (Koshland-Nemethy-Filmer) model [4] has considered finite subunit interactions and proposed a progressive conformational transition of each domain step by step (Fig 1(B)). Both models imply that allosteric processes are closely associated with ligand-driving conformational changes that propagate between the allosterically coupled binding sites. With the development of structural biology, the description of allostery in terms of structure changes was derived [5], and was used to study allosteric proteins such as lactate dehydrogenase [6]. The structure paradigm also leads to the seeking of specific atomic pathway that connects allosteric sites [7–9]. Nevertheless, the discovery of dynamic structure and multiple conformations of proteins, such as multiple orientations of DNA-binding domains of DNA-binding proteins in the absence of DNA [10] and the intermediate conformation of hemoglobin in solution [11], suggests more possibilities beyond the simple two-state models.

The discovery of intrinsically disordered proteins (IDPs) and intrinsically disordered regions (IDRs) has brought a challenge to the conventional “structure-function” paradigm [13–16]. IDPs/IDRs do not have ordered structures in the free state under physiological conditions, but they are important in biological signaling and regulation [17–25]. IDPs/IDRs possess some advantages over ordered proteins [26], such as high specificity coupled with low affinity useful for reversible signaling interaction [27–30], binding to multiple partners [31,32], and rapid turnover allowing sensitive response to environment changing [13,20,33]. Therefore, they play crucial roles in widespread categories of proteins [23], e.g., scaffold proteins [34], RNA and protein chaperones [35], transcription factors [21], regulation of cellular pathways [36], and the recent liquid-liquid phase separations [37]. In particular, IDPs/IDRs were found to be widely involved in allosteric regulation in despite of their lack of ordered structures [38–48]. Representative examples include enzyme aminoglycoside N-(6’)-acetyltransferase II (AAC), which has local unfolding and switching behaviors from positive cooperativity to negative cooperativity upon different temperature [40]; and Doc/Phd toxin-antitoxin system with intrinsic disorder exhibiting complex “conditional cooperativity” character upon different Doc/Phd ratio [41,45].

How can IDPs/IDRs implement allosteric effect under the lack of ordered structures? And why are they so prevalent in allosteric regulation? The answer is related to an emerging new view of allostery based on the general landscape theory of protein structure, where the ligand binding stabilizes specific states and shifts the conformational ensemble [49–51]. The EAM (Ensemble Allostery Model) model used the ensemble view to explain the allostery of IDPs [12,52–55], see Fig 1(C). As an example, it described a two domain system as a four-state ensemble with each domain having ordered (R) and disordered (I) states. The allosteric ligand (A) binds only with the R state of the first domain while the substrate (B) binds only with the R state of the second domain, i.e., the disordered states have no affinity to ligand and substrate. When the interface-interaction free energy between two ordered domains is negative, binding of the ligand A would stabilize the RR state and thus facilitate the binding of the substrate B, resulting in a positive allosteric effect. Similarly, a negative allosteric effect arises when the interface interaction is unfavorable. The EAM model also provided insight in explaining why IDPs/IDRs are so prevalent in allosteric regulation: it was shown that high allosteric intensity is accompanied by high probability of disordered (I) states [12]. However, in investigating the role of protein disorder in allostery, the pristine EAM model considers only the order-disorder (R-I) transition [12], but lacks the order-order (R-T) transition as that in the MWC model for the allostery of ordered proteins. Therefore, with separate EAM or MWC models, it is impossible to determine whether disordered or ordered proteins are more advantageous in allosteric regulation. To get a full view of competition of ordered and disordered proteins in allosteric effect, here we propose a comprehensive ensemble model considering both order-disorder and order-order transitions. In this comprehensive model, the EAM and MWC mechanisms become two pathways for allostery of the system, and thus their role can be quantitatively evaluated.

## Models

### The comprehensive ensemble model

**Fig 2:**
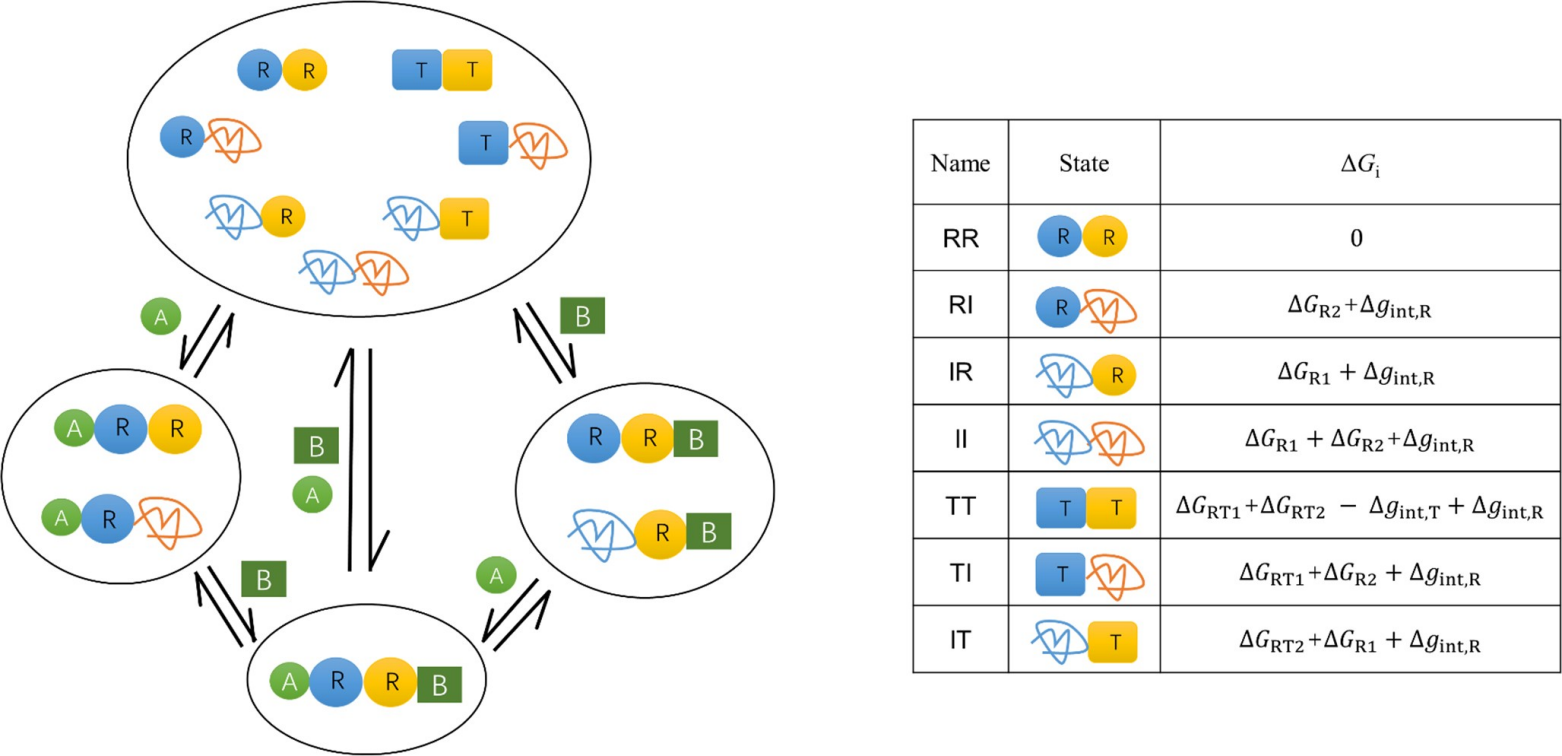
Schematic representation of the proposed comprehensive ensemble model, which compounds the assumptions of both the MWC and the EAM models. Each domain (blue and yellow) can be in R (Relaxed), T (Tensed) and I (Disordered) state. R and T are incompatible as assumed in the MWC model, and thus there are now seven possible combinations for protein states. Similar to the EAM model, the disordered I state of a domain does not have any interface interaction with the adjacent domain, and it does not bind to any ligand or substrate due to the lack of ordered structures. The expressions for the free energy (Δ*G*_*i*_) of each state (relative to RR as the reference state as that in the EAM model) were listed in the absence of ligand and substrate. Δ*G*_R1_ and Δ*G*_R2_ are the free energy of unfolding the R state of each domain, and Δ*g*_int,R_ and Δ*g*_int,T_ are the free energy of breaking the interface interactions in RR and TT, respectively, which were defined in a manner similar to the EAM model. Δ*G*_RT1_ and Δ*G*_RT2_ are the free-energy of the R-T transition for each domain. A is allosteric regulation ligand binding to one (blue) domain, and B is the substrate to the other (yellow) domain. A and B are different molecules, i.e., we consider the heterotropic allosteric effect. To enable both positive and negative allosteric effect for ordered proteins, we consider two binding modes for A: it can only bind to the R state of the blue domain (A-R binding mode) (as depicted here), or can only bind to the T state of the blue domain (A-T binding mode). B always binds only to the R state of the yellow domain.

Our proposed model describes a two-domain protein system (Fig 2). It compounds the assumptions of both the MWC model and the pristine EAM model. Each domain has three states: R (Relaxed), T (Tense) and I (Disordered). To keep consistent with the MWC model, R and T are assumed to be incompatible with each other and thus the combinations “RT” and “TR” are forbidden in the resulting protein states. Similar to the EAM model, the I state of a domain is disordered and does not have any interface interaction with the adjacent domain, and it does not bind to any ligand or substrate due to the lack of ordered structures. As a result, there are seven possible combinations for protein states, which are listed in Fig 2 with the formula of their free energy Δ*G*_*i*_ in the absence of ligand and substrate. Six free energy parameters (Δ*G*_R1_, Δ*G*_R2_, Δ*g*_int,R_, Δ*g*_int,T_, Δ*G*_RT1_, Δ*G*_RT2_) are basic parameters of the model, determining the ensemble distribution. The corresponding probability *P*_*i*_ of each state is related to their free energy Δ*G*_*i*_ as $P_{i}=e^{-\Delta G_{i}/RT}/Q$ where *Q* is the sum of statistical weights as $Q=\sum_{i}e^{-\Delta G_{i}/RT}$. The substrate B binds only to the R state of one (yellow) domain. The allosteric ligand A binds to the other (blue) domain but there are two binding modes: in the A-R binding mode A binds only to the R state of the blue domain (as depicted in Fig 2), while it is the A-T binding mode when A binds only to the T state of the blue domain. The two binding modes are taken into account here to enable both positive and negative allosteric effects for ordered proteins (MWC mechanism), making a comparison between the roles of ordered and disordered proteins possible. For example, if we look at a subsystem consisting of RR and TT states, binding of A in the A-R binding mode increases the fraction of the RR state and thus enhances the subsequent binding of B (activation), while that in the A-T binding mode weakens the binding of B (inhibition).

### Definitions of contribution of ordered and disordered protein pathways to allostery of the comprehensive ensemble model

Adding allosteric ligand A to the system results in a redistribution of the protein ensemble probabilities, i.e., population shift [56]. The allosteric effect is directly related to probability variation of the states that can bind substrate B due to the adding of A. There were various ways to measure the allosteric response [1,12,57,58]. Following the EAM model [12], here we define the allosteric coupling response (*CR*) as

$$CR=\frac{P_{X,[A]}-P_{X,[A]=0}}{-\Delta g_{\mathrm{Lig}.A}/RT}$$ (1)

to quantitatively measure the allosteric intensity for a given system. Here, X denotes the states that can bind B, so *P*_X,[A]_ is the probability of states that can bind B when there exists ligand A, and *P*_X,[A] = 0_ is the probability when A is absent. The influence of other measurements of allostery will be discussed below. In the comprehensive ensemble model proposed here, for the A-R binding mode we have *P*_X,[A]_ = *P*_ARR_ + *P*_RR_ + *P*_IR_, and for the A-T binding mode we have *P*_X,[A]_ = *P*_RR_ + *P*_IR_. Δ*g*_Lig.A_ is the stabilizing free energy of adding ligand A for the states that can bind A, which is determined as:

$$\Delta g_{\mathrm{Lig}.A}=-RT\ln(1+K_{a,A}\times [A]),$$ (2)

where *K*_a,A_ is the intrinsic equilibrium constant of the binding reaction for A. For example, in the A-R binding mode, *K*_a,A_ is the association constant for the reactions A + RR = ARR and A + RI = ARI, which gives the equilibrium distributions:

$$\left\{ \begin{array}{l} \left[\mathrm{RR}]+[\mathrm{ARR}]=[\mathrm{RR}]+K_{a,A}[A][\mathrm{RR}]=e^{-\Delta g_{\mathrm{Lig},A}/RT}[\mathrm{RR}\right]\\ \left[\mathrm{RI}]+[\mathrm{ARI}]=[\mathrm{RI}]+K_{a,A}[A][\mathrm{RI}]=e^{-\Delta g_{\mathrm{Lig},A}/RT}[\mathrm{RI}\right] \end{array}\right.,$$ (3)

clearly demonstrating the nature of the stabilizing free energy Δ*g*_Lig,A_. In our study, we fixed Δ*g*_Lig.A_ = −3.0 kcal/mol at a physiological temperature of *T* = 310.15 K as in the EAM model unless otherwise specified.

**Fig 3:**
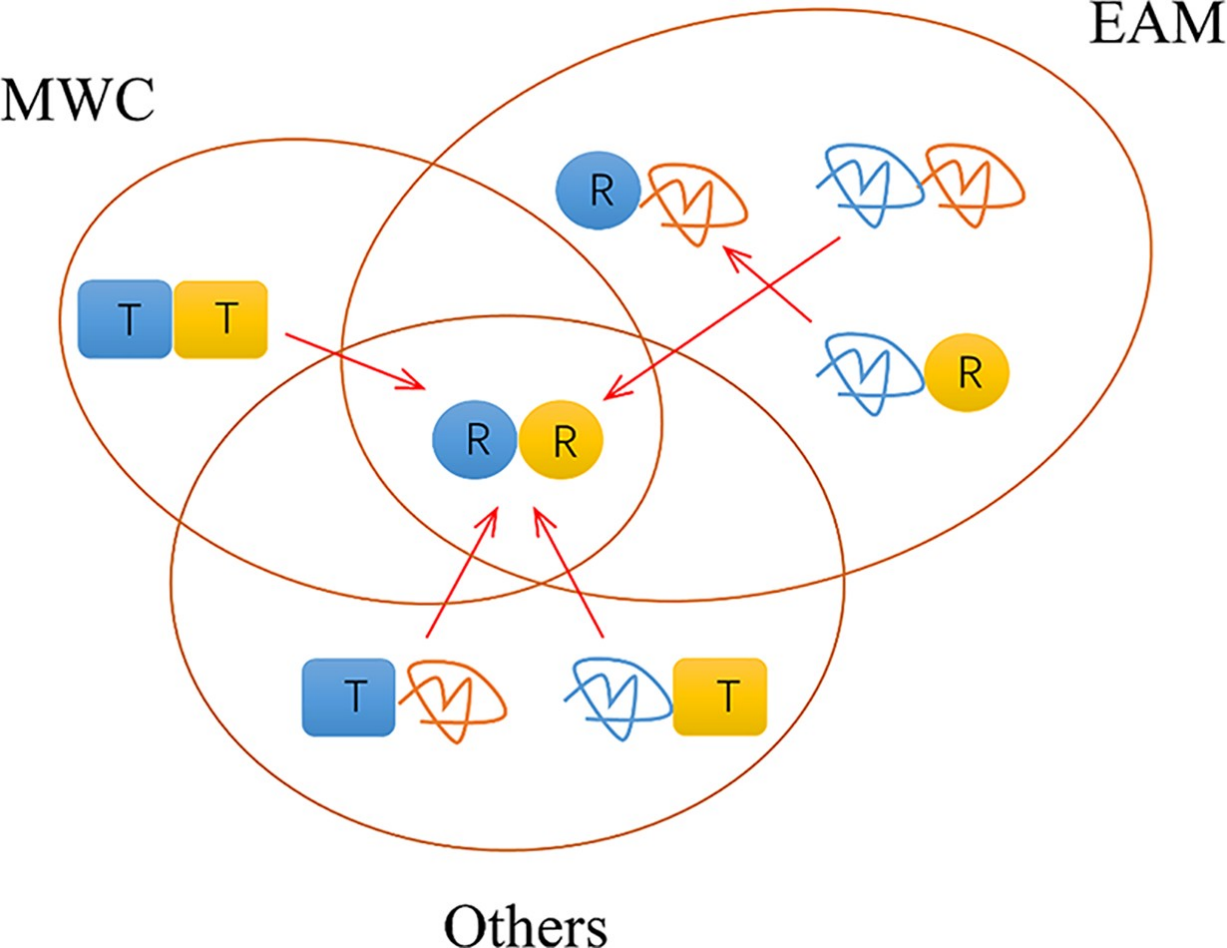
Three allosteric subsystems/pathways in the comprehensive ensemble model. The arrows indicate the population shifts caused by ligand binding which are capable of affecting the substrate binding (see text for details). The A-R binding mode is assumed here. The contribution ratios of pathways to the allostery of the comprehensive system are given in Eqs (4–7).

Because the comprehensive model includes all the states of the MWC model and the EAM model, we can also view the comprehensive system consisting of three subsystems: the MWC subsystem, the EAM subsystem and the Others subsystem (Fig 3). If the allostery occurs via the order-order transition within the MWC subsystem (involving the states RR and TT), it was classified into the MWC pathway. If the allostery occurs via the disorder-order transition within the EAM subsystem (RR, RI, IR and II), it was classified into the EAM pathway. Similar definition applies for the Others pathway involving RR and the remaining states (TI and IT) neglected in the MWC and EAM mechanisms. Binding of the allosteric ligand A in the A-R binding mode causes population shifts of $\left[\bar{R}*]\to [R*\right]$, where $\bar{R}$ represents the non-R domain states (T and I), while * represents any domain states (R, T, I). On the other hand, only the population shifts as $\left[*\bar{R}]\to [*R\right]$ could affect the binding of the substrate B. Overall, the population shifts caused by ligand binding which are capable of affecting the substrate binding includes “II→RR”, “TT→RR”, “TI→RR”, “IT→RR” and “IR→RI” (indicated by arrows in Fig 3), which all occurs within the decomposed subsystems here. Therefore, the classification of three pathways is complete and there is no allosteric communication among them. The MWC pathway contains allosteric order-order transition, and the EAM pathway contains allosteric order-disorder transition, while the Others pathway is a mixed one with both order-order and order-disordered transitions, e. g., “TI→RR” is composed of an order-order “T→R” in one domain and an order-disorder “I→R” in the second domain.

The allosteric coupling response (*CR*) of each subsystem can be defined and calculated separately, i.e., to assume each subsystem exists alone. Take the MWC subsystem as an example (under A-R binding mode), we have

$$CR_{\mathrm{MWC}}=\frac{P_{\mathrm{RR}+\mathrm{ARR},[A]}^{\left(\mathrm{MWC}\right)}-P_{\mathrm{RR},[A]=0}^{\left(\mathrm{MWC}\right)}}{-\Delta g_{\mathrm{Lig},A}/RT},$$ (4)

where the superscript “(MWC)” indicates that the related probabilities of states are defined (normalized) within the MWC subsystem, i.e., $P_{RR+ARR,[A]}^{\left(MWC\right)}=\frac{P_{RR}+P_{ARR}}{P_{RR}+P_{ARR}+P_{TT}}$ and $P_{RR,[A]=0}^{\left(MWC\right)}=\frac{P_{RR}}{P_{RR}+P_{TT}}$. Similarly, *CR* for the EAM subsystem and the Other subsystem are determined by

$$\left\{ \begin{array}{l} CR_{\mathrm{EAM}}=\frac{P_{\mathrm{RR}+\mathrm{ARR}+\mathrm{IR},[A]}^{\left(\mathrm{EAM}\right)}-P_{\mathrm{RR}+\mathrm{IR},[A]=0}^{\left(\mathrm{EAM}\right)}}{-\Delta g_{\mathrm{Lig},A}/RT}\\ CR_{\mathrm{Others}}=\frac{P_{\mathrm{RR}+\mathrm{ARR},[A]}^{\left(\mathrm{Others}\right)}-P_{\mathrm{RR},[A]=0}^{\left(\mathrm{Others}\right)}}{-\Delta g_{\mathrm{Lig},A}/RT} \end{array}\right.,$$ (5)

where $P_{RR+ARR+IR,[A]}^{\left(EAM\right)}=\frac{P_{RR}+P_{ARR}+P_{IR}}{P_{RR}+P_{ARR}+P_{RI}+P_{ARI}+P_{IR}+P_{II}}$ and $P_{RR+ARR,[A]}^{\left(Others\right)}=\frac{P_{RR}+P_{ARR}}{P_{RR}+P_{ARR}+P_{TI}+P_{IT}}$. It is noted that $P_{RR+ARR,[A]}^{\left(MWC\right)}\neq P_{RR+ARR,[A]}^{\left(Others\right)}$ since they are normalized within different subsystems. With a set values of the basic parameters (Δ*G*_R1_, Δ*G*_R2_, Δ*g*_int,R_, Δ*g*_int,T_, Δ*G*_RT1_, Δ*G*_RT2_), it is thus straightforward to calculate the probabilities of all the states with and without ligand A, as well as *CR* for the whole system (*CR*_tot_) and subsystems (*CR*_MWC_, *CR*_EAM_, *CR*_Others_). The contribution of a pathway to the total allostery of the comprehensive system depends not only on *CR* of the corresponding subsystem, but also on the proportion of the subsystem states in the whole system. Therefore, the contribution ratio of the MWC pathway to the allostery of the comprehensive system is approximately defined as:

$$Weight_{\mathrm{MWC}}=\min(P_{\mathrm{RR}+\mathrm{TT},[A]=0},P_{\mathrm{RR}+\mathrm{ARR}+\mathrm{TT},[A]})\times CR_{\mathrm{MWC}}/CR_{\mathrm{tot}}.$$ (6)

It stands for the weight of the MWC pathway in the allosteric effect. When there are only RR and TT states before adding ligand A, the comprehensive model degenerates to the MWC model and Eq (6) gives *Weight*_MWC_ = 1. Similarly, for the EAM and the Others pathways, we have:

$$\left\{ \begin{array}{l} Weight_{\mathrm{EAM}}=\min(P_{\mathrm{RR}+\mathrm{RI}+\mathrm{IR}+\mathrm{II},[A]=0},P_{\mathrm{RR}+\mathrm{ARR}+\mathrm{RI}+\mathrm{ARI}+\mathrm{IR}+\mathrm{II},[A]})\times CR_{\mathrm{EAM}}/CR_{\mathrm{tot}}\\ Weight_{\mathrm{Others}}=\min(P_{\mathrm{RR}+\mathrm{TI}+\mathrm{IT},[A]=0},P_{\mathrm{RR}+\mathrm{ARR}+\mathrm{TI}+\mathrm{IT},[A]})\times CR_{\mathrm{Others}}/CR_{\mathrm{tot}} \end{array}\right..$$ (7)

It is noted that *Weight*_MWC_, *Weight*_EAM_ and *Weight*_Others_ are metrics for three pathways’ contributions to allosteric effect of the comprehensive system, but the sum of them is not necessarily equal to 1.0 although the deviation is usually small. Related equations under the A-T binding mode can be found in Supporting Information.

## Results

### Limits for the maximal allosteric response

**Fig 4:**
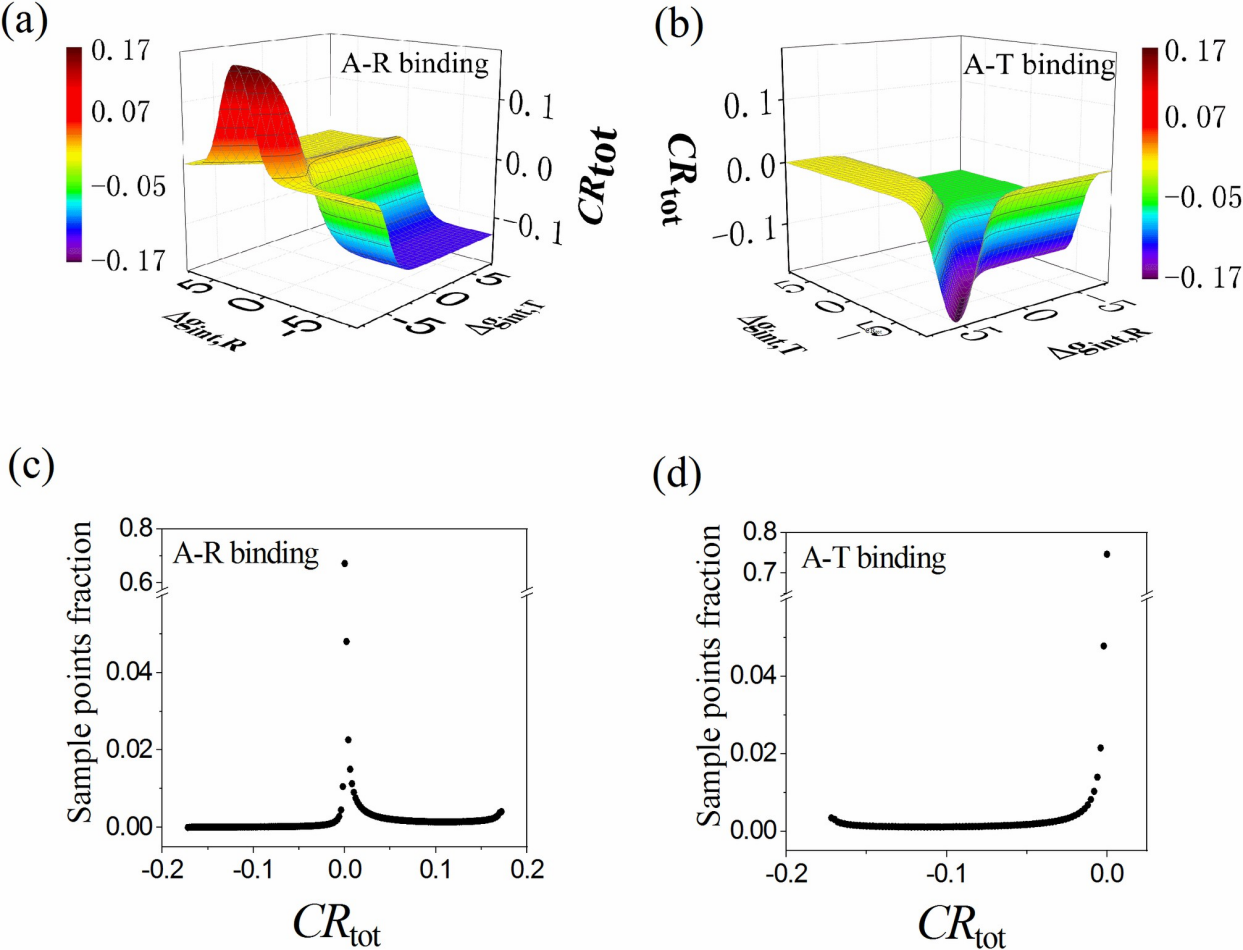
The allosteric coupling response (*CR*) of the comprehensive ensemble model. (a,b) *CR* as a function of Δ*g*_int,R_ and Δ*g*_int,T_ (in kcal/mol) when the other parameters are fixed (chosen to produce notable allostery) as: Δ*G*_R1_ = −1.0, Δ*G*_R2_ = 1.3, Δ*G*_RT1_ = 1.0, Δ*G*_RT2_ = 3.0 (all in units of kcal/mol). Note that for A-T binding mode there is no activated allosteric effect. (c,d) Distribution of *CR* when the stability free-energy parameters (Δ*G*_R1_, Δ*G*_R2_, Δ*G*_RT1_, Δ*G*_RT2_, Δ*g*_int,R_, Δ*g*_int,T_) vary randomly with an equal probability density between −8 and +8 kcal/mol. The A-R binding mode is adopted in (a,c) and the A-T binding mode is adopted in (b,d) with Δ*g*_Lig,A_ = −3 cal/mol.

With a given set of parameters for protein state stability (Δ*G*_R1_, Δ*G*_R2_, Δ*G*_RT1_, Δ*G*_RT2_, Δ*g*_int,R_, Δ*g*_int,T_) and protein-ligand interaction (Δ*g*_Lig,A_) of the proposed comprehensive ensemble model, we can calculate the ensemble distribution, the allosteric coupling response (*CR*) and the contributions of different pathways with the formulism described above. *CR* as a function of Δ*g*_int,R_ and Δ*g*_int,T_ is shown in Fig 4A and 4B as a case example when the other parameters are fixed. It reveals that combination of Δ*g*_int,R_ and Δ*g*_int,T_ is required to maximize the allosteric effect. Under the A-R binding mode, the model can afford both positive (*CR* > 0) and negative (*CR* < 0) allosteric effects, while there is only negative effect under the A-T binding mode. The achieved highest *CR* is about 0.17. To have a global inspection on the occurring probability of allostery, we assume the stability free-energy parameters (Δ*G*_R1_, Δ*G*_R2_, Δ*G*_RT1_, Δ*G*_RT2_, Δ*g*_int,R_, Δ*g*_int,T_) vary randomly between [–8, +8] kcal/mol, and determine the distribution of *CR* for two binding modes with Δ*g*_Lig,A_ = −3 cal/mol (Fig 4C and 4D). For the majority of parameter sets, the resulting allostery is weak, giving a sharp peak at *CR* = 0 for both binging modes (Fig 4C and 4D). Actually, only 6.3% of parameter sets produce |*CR*| > 0.1 under the A-R binding mode. Remarkably, *CR* has the boundaries at around ±0.172. In other words, no matter how the state stabilities of protein are optimized, it is impossible to achieve a *CR* value higher than 0.172.

The boundary limits of *CR* can be well explained in an analytic way. Take the MWC model as a simplified example, there are two states (RR and TT state) with only one stability parameter (Δ*G*_*i*_ ≡ *G*_RR_−*G*_TT_), which determines the probability of RR state without ligand to be:

$$P_{\mathrm{RR}}=\frac{e^{-\Delta G_{i}/RT}}{e^{-\Delta G_{i}/RT}+1}=1-P_{\mathrm{TT}}.$$ (8)

**Fig 5:**
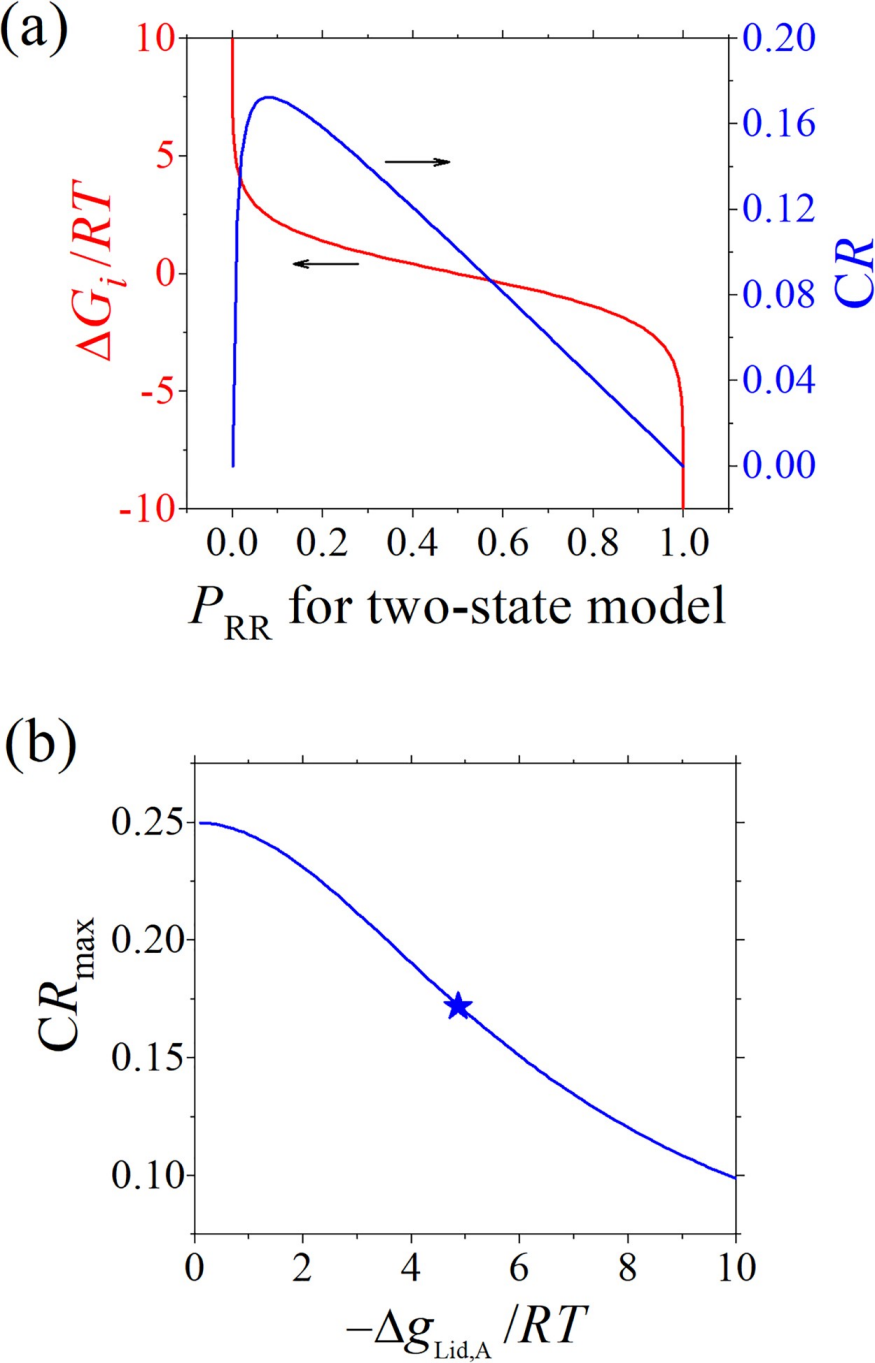
Limits of CR. (a) The relations among *P*_RR_, Δ*G*_*i*_ and *CR* for the MWC model with two states, plotted according to Eqs 8 and 9. (b) The *CR* maximum (via optimizing state stabilities of proteins) as a function of −Δ*g*_Lig,A_/*RT*, plotted according to Eq (10) which is valid for the comprehensive ensemble model as well as the MWC and EAM models. The star represents the data point for Δ*g*_Lig,A_ = −3 kcal/mol and *T* = 310.15 K used in other figures.

*CR* can then be written as a function of *P*_RR_ and Δ*g*_Lig,A_ as

$$\begin{array}{l} CR=\left[\frac{P_{\mathrm{RR}}e^{-\Delta g_{\mathrm{Lig},A}/RT}}{P_{\mathrm{TT}}+P_{\mathrm{RR}}e^{-\Delta g_{\mathrm{Lig},A}/RT}}-P_{\mathrm{RR}}\right]\cdot \frac{1}{-\Delta g_{\mathrm{Lig},A}/RT}\\ \;=\left[\frac{P_{\mathrm{RR}}e^{-\Delta g_{\mathrm{Lig},A}/RT}}{1-P_{\mathrm{RR}}+P_{\mathrm{RR}}e^{-\Delta g_{\mathrm{Lig},A}/RT}}-P_{\mathrm{RR}}\right]\cdot \frac{1}{-\Delta g_{\mathrm{Lig},A}/RT} \end{array}$$ (9)

under the A-R binding mode. The relations among *P*_RR_, Δ*G*_i_ and *CR* are plotted in Fig 5(A) for Δ*g*_Lig,A_ = −3 kcal/mol. *CR* is equal to 0 at either *P*_RR_ = 0 or *P*_RR_ = 1, i.e., too stable and too unstable RR state are unfavorable to allostery. *CR* reaches its maximum of about 0.172 at *P*_RR_ = 0.081. *P*_RR_ depends on Δ*G*_*i*_ in a switch-like manner. A great many Δ*G*_*i*_ values give *P*_RR_ close to 0 or 1, and result in small *CR* and weak allostery. This provide a clue in understanding the dominant peak at *CR* = 0 in Fig 4C and 4D. Based on Eq (9), the maximization of *CR* can be solved analytically with $\frac{\partial CR}{\partial P_{\mathrm{RR}}}=0$ to give

$$CR_{\max}=\frac{{\left(e^{-\Delta g_{\mathrm{Lig},A}/2RT}-1\right)}^{2}}{\left(-\Delta g_{\mathrm{Lig},A}/RT\right)\left(e^{-\Delta g_{\mathrm{Lig},A}/RT}-1\right)}$$ (10)

at the optimized *P*_RR_ as

$$P_{\mathrm{RR}}^{\left(\mathrm{opt}.\right)}=\frac{e^{-\Delta g_{\mathrm{Lig},A}/2RT}-1}{e^{-\Delta g_{\mathrm{Lig},A}/RT}-1}.$$ (11)

Eq (10) keeps valid for the comprehensive ensemble model (see Supporting Information). *CR*_max_ is plotted in Fig 5(B) as a function of −Δ*g*_Lig,A_/*RT* (note that Δ*g*_Lig,A_ < 0). It decreases with increasing −Δ*g*_Lig,A_/*RT*, and reaches a value of 0.172 at Δ*g*_Lig,A_ = −3 kcal/mol and *T* = 310.15 K, being consistent with the observation in Fig 4. Eq (10) gives an analytical result for the limits of *CR* when the state stabilities of protein are optimized, and would be useful in studying the allosteric capacity of proteins.

### The weight of MWC pathway is significantly higher than that of EAM pathway

**Fig 6:**
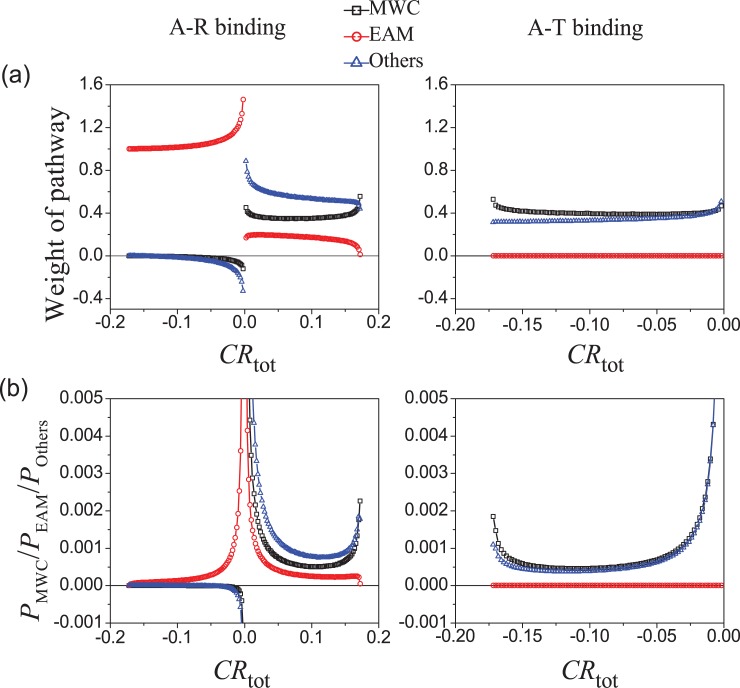
Contributions of three pathways (MWC, EAM, Others) in the comprehensive ensemble model. The stability free-energy parameters (Δ*G*_R1_, Δ*G*_R2_, Δ*G*_RT1_, Δ*G*_RT2_, Δ*g*_int,R_, Δ*g*_int,T_) vary randomly with an equal probability density between −8 and +8 kcal/mol, resulting in different systems (samples). (a) The average weights of pathways as functions of *CR*_tot_. The pathway weights of a system (sample) are calculated based on Eqs 6 and 7. (b) The capacity of three pathways for allostery, being calculated with Eq (12). The A-R binding mode is adopted in left panels and the A-T binding mode is adopted in right panels with Δ*g*_Lig,A_ = −3 kcal/mol. It is noted that a large portion of samples practically have *CR* = 0 and the pathway contributions are ill-defined with Eqs 6 and 7, which are thus ignored.

The weights of three pathways (MWC, EAM and Others) in the allostery of the comprehensive system are numerically analyzed when the stability free-energy parameters (Δ*G*_R1_, Δ*G*_R2_, Δ*G*_RT1_, Δ*G*_RT2_, Δ*g*_int,R_, Δ*g*_int,T_) vary randomly between [–8, +8] kcal/mol. The resulting average weights are shown in Fig 6(A) as functions of *CR*. For positive allosteric effect (*CR* > 0), the weight of the MWC pathway is much larger than the EAM one, indicating the MWC pathway holds an advantage over the EAM pathway in this case. For negative allosteric effect, *CR* under the A-R binding mode mainly comes from the EAM pathway, while under the A-T binding mode *CR* mainly comes from the MWC and Others pathways. The reason is that when A binds with R, in the MWC subsystem the decrease of RR state is not allowed and thus its weight is almost zero or even negative based on Eq (6), while an IR→RI transition of EAM pathway dominates the negative allosteric response. On the other hand, when A binds with T, it has no effect in the state distribution in the EAM subsystem thus its weight is always zero.

The capacity of the MWC or the EAM pathway for allostery depends on not only their weights in a comprehensive system (Fig 6(A)) but also the possibility of the system to afford an allosteric effect (*P*(*CR*), see Fig 4(B)). Therefore, the possibility for allosteric effect with *CR* undertaken by the MWC pathway can be calculated as

$$P_{\mathrm{MWC}}(CR)=\bar{Weight_{\mathrm{MWC}}}(CR)\times P(CR).$$ (12)

It describes the probability of a randomly chosen parameter set to possess an allosteric effect *CR* via the MWC pathway. Formula for the EAM and Others pathways can be similarly written. The calculated results are shown in Fig 6(B). *P*_MWC_(*CR*) and *P*_Others_(*CR*) has sharp peaks near the positive allostery limit *CR*_max_ in the A-R binding mode and near the negative allostery limit −*CR*_max_ in the A-T binding mode, which will be discussed in detail below. More importantly, if we take a simplified approach by adding curves in the A-R and A-T binding modes for each pathway, *P*_MWC_(*CR*) is much larger than *P*_EAM_(*CR*) for strong allosteric effects. Therefore, the MWC pathway is more important in allosteric effects than the EAM pathway based on the comprehensive ensemble model.

### Probability of strong allostery first increases and then decreases when the Δ*G*_*i*_ range increases

**Fig 7:**
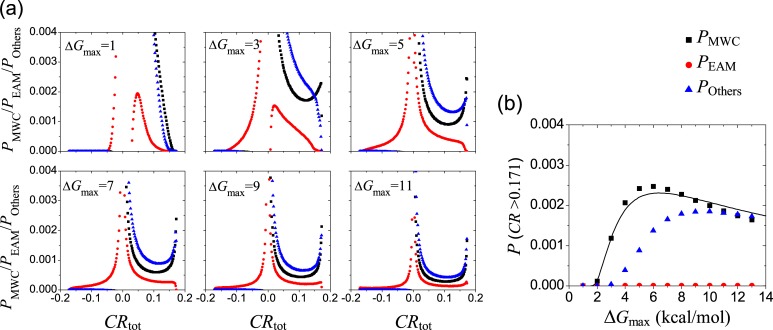
The influence of the variation range (Δ*G*_max_, in a unit of kcal/mol) for free-energy parameters (Δ*G*_R1_, Δ*G*_R2_, Δ*G*_RT1_, Δ*G*_RT2_, Δ*g*_int,R_, Δ*g*_int,T_) of the comprehensive ensemble model with the A-R binding mode. (a) The possibilities for three pathways [calculated with Eq (12)] obtained at different [–Δ*G*_max_, +Δ*G*_max_] range. (b) The probabilities of *CR* > 0.171 as a function of Δ*G*_max_.

The distribution of allostery and pathway contribution were investigated above when the free-energy parameters (Δ*G*_R1_, Δ*G*_R2_, Δ*G*_RT1_, Δ*G*_RT2_, Δ*g*_int,R_, Δ*g*_int,T_) of the comprehensive model vary randomly in a range of [–8, +8] kcal/mol. The results may change under a different range. In Fig 7(A), the possibilities for an allosteric effect to occur with *CR* undertaken by three pathways are plotted under various variation range [–Δ*G*_max_, +Δ*G*_max_] of the free-energy parameters. The sharp peaks of *P*_MWC_ and *P*_Others_ near the positive allostery limit (*CR*_max_ = 0.172) observed previously are absent when the variation range (Δ*G*_max_) is small, e.g., Δ*G*_max_ = 1 kcal/mol. In Fig 7(B), the probabilities of *CR* > 0.171 for three pathways are plotted as a function of Δ*G*_max_. It clearly shows that the MWC and the Others pathways have a similar tendency: it first equals to zero before a critical Δ*G*_max_ (which is smaller for the MWC pathway), then increases quickly, and finally decreases slowly.

The feature observed in Fig 7 can be qualitatively explained based on the simplified two-state model (Fig 5). The maximal *CR* is achieved at *P*_RR_ = 0.081, which corresponds to a free energy difference of Δ*G*_*i*_(≡*G*_RR_−*G*_TT_) = 1.6 kcal/mol. When the variation range of the free-energy parameters is small, the resulting Δ*G*_*i*_ cannot reach the optimized value for the maximal *CR*, giving the zero value in Fig 7(B) and the absence of the sharp peak near *CR*_max_ in the panel with Δ*G*_max_ = 1 kcal/mol in Fig 7(A). When the variation range of the free-energy parameters is large enough, although the optimized value of Δ*G*_*i*_ can be always satisfied at some values of parameter sets, the total number of possible values increases with the variation range, and thus the probability of maximal *CR*, defined as the ratio between the number of optimized parameter value sets to that of the total number, would decreases with increasing the variation range as observed in Fig 7(B).

### Two-state transition is the main mechanism for strong allostery

The comprehensive ensemble model includes seven states and three subsystems/pathways. How do they coordinate in fulfilling the allosteric effect? For example, do the pathways repeal each other in a system? How many states play significant role in a system? Here, we investigate the interplay between different states and different subsystems/pathways in the allosteric process.

**Fig 8:**
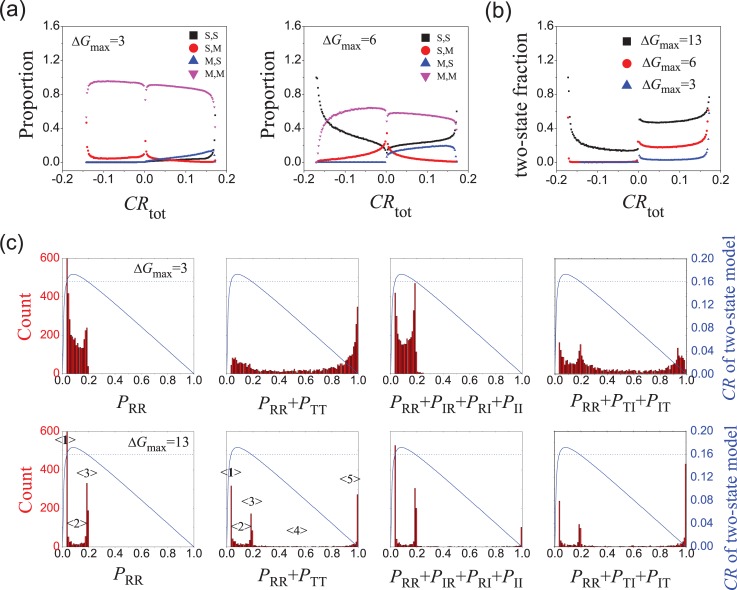
Interplay between different states and subsystems/pathways in the comprehensive ensemble model. **The A-R binding mode is adopted.** (a) Proportion of four categories [single subsystem with single pathways (S,S), single subsystem with mixing pathways (S,M), mixing subsystems with single pathways (M,S), and mixing subsystems with mixing pathways (M,M)] in systems with *CR*. (b) The proportion of systems with two-state transition. (c) Distribution of *P*_RR_ and state probability of three subsystems (*P*_RR_ + *P*_TT_ for the MWC pathway, *P*_RR_ + *P*_IR_ + *P*_RI_ + *P*_II_ for the EAM pathway, and *P*_RR_ + *P*_TI_ + *P*_IT_ for the Other pathway) for systems with *CR* ≈ 0.16. Δ*G*_max_ = 3 or 13 kcal/mol. The theoretical *CR* ~ *P*_RR_ curve (blue line) for the two-state model is also plotted using Eq (9). The horizontal dashed line represents *CR* = 0.16.

To measure the mixing extend of subsystems and pathways, we classify each system case (with a certain set of Δ*G*_*i*_ values) into one of four categories: single subsystem with single pathway (S,S), single subsystem with mixing pathways (S,M), mixing subsystems with single pathway (M,S), and mixing subsystems with mixing pathways (M,M). If the sum of state probability for any subsystem is larger than 0.99 before and after adding ligand, it is classified into single subsystem; otherwise it belongs to mixing subsystem. Single pathway is defined for the case where the weight of one pathway is larger than 0.99 and the absolute value of weights for other pathways are less than 0.01; otherwise it belongs to mixing pathway. For example, if a system only contain RR and TT states, then it simply belongs to the (S,S) category. The results are shown in Fig 8(A). When the variation range (Δ*G*_max_) of free-energy parameters is small, mixing subsystems with mixing pathways (M,M) dominate in most cases. But when Δ*G*_max_ is larger, the proportion of single subsystem with single pathway (S,S) increases while the (M,M) type decreases. More importantly, the (S,S) proportion increases with increasing |*CR*|. The system tends to behave as pure subsystem with pure pathway mechanism at strong allostery.

A clearer angle of view is to look at the proportion of systems that implement allostery via a simple mechanism of two-state transition. Here we specify a system to have two-state transition mechanism if the probability sum of two certain states of the given system is larger than 0.99 both before and after binding with ligand A. Possible two-state transition for positive allosteric effect includes “II→RR”, “TT→RR”, “TI→RR” and “IT→RR”. For negative allosteric effect, the only possible two-state transition is “IR→RI”. The proportion of systems with simple two-state transition is shown in Fig 8(B). With larger Δ*G*_max_, the proportion of two-state transition is higher. The proportion has a sharp peak at ±*CR*_max_. Therefore, two-state transition is the major mechanism for strong allosteric even in the comprehensive ensemble model.

The existence of two-state transition and single subsystem/pathway are also reflected in the state distribution patterns. The distributions of RR and states of three subsystems are shown in Fig 8(C) for systems with *CR* ≈ 0.16. The distribution of *P*_RR_ has two obvious peaks labeled with **<1>** and **<3>**. In Fig 8(C) we also plot the theoretical *CR* ~ *P*_RR_ curve for the two-state model for convenience's sake. The crossing points between the *CR* ~ *P*_RR_ curve and the horizontal line of *CR* = 0.16 give the *P*_RR_ values to achieve an allosteric effect of *CR* = 0.16 in the two-state model. The obtained *P*_RR_ values of the crossing points coincide with the peak position at **<1>** and **<3>** of the simulated *P*_RR_ distribution, suggesting that the strong allostery (with *CR* = 0.16) of the comprehensive model mainly occurs in a two-state model mechanism (note that RR exists in all possible two-state transition for positive *CR* including “II→RR”, “TT→RR”,“TI→RR” and “IT→RR”). There is also some nonzero *P*_RR_ distribution (**<2>**) between two peaks, which is expected to have *CR* higher than 0.17 in the two-state model. The reason for that is the introducing of additional IR and RI population would decrease *CR* (see Supplementary Material). It also explains the intriguing result that there is no distribution outside **<1>**&**<3>**, for *P*_RR_ outside cannot give *CR* as big as 0.16. When Δ*G*_max_ increases to 13 kcal/mol, *P*_RR_ distribution enriches at **<1>**&**<3>** and reduces at **<2>**, suggesting an enrichment of two-state transition mechanism. Similarly, for the distribution of the MWC pathway states, the *P*_RR_ + *P*_TT_ peaks at **<1>**&**<3>** correspond to the systems dominated by other pathways (EAM or Others) so that *P*_RR_ + *P*_TT_ = *P*_RR_ and the peak positions are identical to that for *P*_RR_. At **<5>**, *P*_RR_ + *P*_TT_ = 1 corresponds to the systems dominated by the MWC pathway. **<2>** and **<4>** mean hybridized cases. Results for the population distribution of the EAM and Others subsystems are similar. They confirm that strong allostery in the comprehensive ensemble mode is dominated by single pathway and the two-state transition mechanism.

### The conclusions are similar under other measures of allostery and interaction schemes

**Fig 9:**
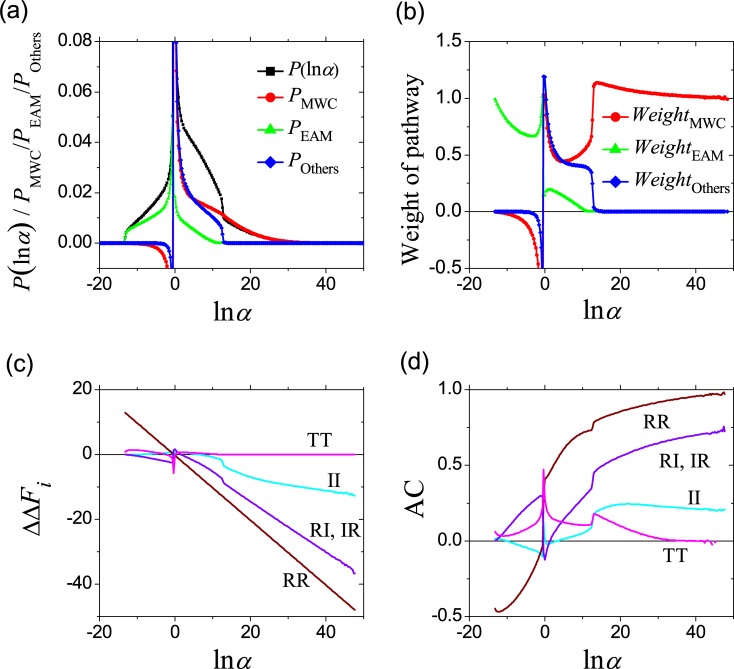
Analysis results with other measures of allostery. (a) Distribution of ln*α* where *α* is the thermodynamic allosteric efficacy defined in Eq (13), as well as the capacity of three pathways. (b) The average weights of pathways as functions of ln*α*. (c) The average thermodynamic coupling function [ΔΔ*F*_*i*_, as defined in Eq (15), and in a unit of *RT*] at some states as functions of ln*α*. (d) The average normalized allosteric coupling [*AC*, as defined in Eq (16)] of some states as functions of ln*α*. The variation range (Δ*G*_max_) for free-energy parameters is 8 kcal/mol.

In addition to the allosteric coupling response (*CR*) we adopted above, there were various ways to measure the allosteric response [1,57,58]. For example, a widely-used one is the thermodynamic allosteric efficacy *α* defined as [57]

$$\alpha =\frac{K_{bound}}{K_{unbound}},$$ (13)

where *K*_bound_ and *K*_unbound_ are the equilibrium constants for the binding with substrate B when the protein is bound or unbound to the allosteric ligand A, respectively. For the A-R binding mode of the current comprehensive ensemble model, *α* can be calculated as

$$\alpha =\frac{P_{RR}}{P_{R*}}/\frac{P_{*R}}{P_{**}},$$ (14)

where * indicates the summation over all possible domain states, e.g., *P*_R*_ = *P*_RR_ + *P*_RI_. Different from *CR* which counts the probability difference and thus arbitrarily penalizes the allosteric stabilization of very low probability states (for example, a *P* = 0.0001 state becoming a *P* = 0.01 state would be considered “weak”), *α* counts the fold-change in probabilities and is directly related to thermodynamic energy cycle of allostery [57]. To clarify whether the choice of allosteric measure would affect the conclusions, we examine the distribution of *α* and the contributions from three pathways in Fig 9A and 9B. For positive allosteric effect (ln*α* > 0), the distribution probability decreases with increasing *α*. More importantly, strong allostery is dominantly contributed by the MWC pathway. In other words, the advantage of MWC pathway is even more prominent when *α* is used instead of *CR* to measure allostery.

The pristine EAM model mainly describes the order-disorder transition [12]. It was later extended to become a general two-state model of allostery applicable for any system exhibiting conformational change (e.g., IDPs, folded proteins, or combinations thereof) [59,60]. The comprehensive ensemble model proposed in this study can be regarded as a further generalization to the three-state case where we combined the insight of the pristine EAM model on order-disorder transition (the lack of interface interaction for I) and that of the MWC model on order-order transition (the incompatible interface between R and T). Actually, the latter point is vital for the achievement of allostery. This can be well demonstrated by the thermodynamic coupling function introduced by LeVine *et al*. [58,61]:

$$\Delta \Delta F(x,y)=-RTln\left(\frac{p(x,y)}{p(x)p(y)}\right)$$ (15)

in describing the allostery landscape, where *x* and *y* represents the state of the allosteric and the functional sites, respectively, i.e., the state of the first domain and the second domain. For a simplified case where all free energy parameters are set to 0, we have *P*_RR_ = *P*_IR_ = *P*_RR_ = *P*_II_ = *P*_TT_ = *P*_TI_ = *P*_IT_ = 1/7, *P*_R*_ = P_*R_ = *P*_T*_ = P_*T_ = 2/7 and *P*_I*_ = P_*I_ = 3/7, so it yields that (in unit of *RT*) $\Delta \Delta F_{RR}=\Delta \Delta F_{TT}=-ln\left[\frac{1}{7}/{\left(\frac{2}{7}\right)}^{2}\right]\approx -0.56,\;\Delta \Delta F_{RI}=\Delta \Delta F_{IR}=-ln\left[\frac{1}{7}/\left(\frac{2}{7}∙\frac{3}{7}\right)\right]\approx -0.15$ and $\Delta \Delta F_{II}=-ln\left[\frac{1}{7}/{\left(\frac{3}{7}\right)}^{2}\right]\approx 0.25$. This may explain why the model seems to utilize the order-order pathway more often than the order-disorder one–it is a natural result of fully excluding the RT and TR states. Actually, under the A-R binding mode, *α* defined in Eq (14) is related to ΔΔ*F* in Eq (15) as $ln\alpha =-\frac{\Delta \Delta F_{RR}}{RT}$. A numerical result in Fig 9(C) confirms such a prediction. ΔΔ*F* has a natural normalization when *x* and *y* are discrete, and a normalized allosteric coupling was proposed by LeVine *et al*. as [58]:

$$AC(x,y)=-\frac{\Delta \Delta F(x,y)}{\Delta \Delta F_{max}(x,y)}=\frac{ln(p(x)p(y))}{ln(p(x,y))}-1,$$ (16)

which ranges between 1 and −1 and its magnitude describes what fraction of the maximal allostery is contributing to the free energy of the joint state. Analysis on *AC* is given in Fig 9(D). *AC* of RR is close to 1, indicating its central role in allosteric response.

**Fig 10:**
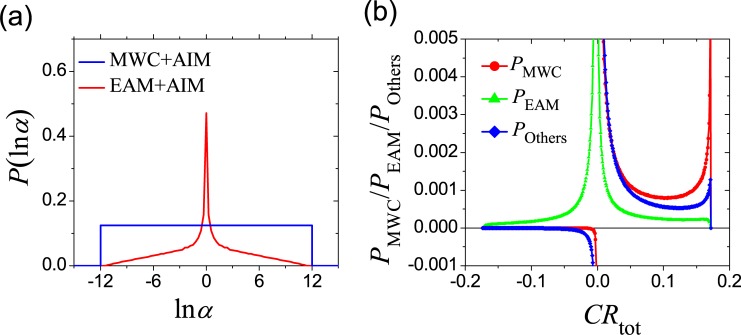
The properties under Ising-like interface interactions. (a) Distribution of ln*α* when the Ising-like interface interactions of the Allosteric Ising Model (AIM) were introduced to combine with the MWC and EAM models. The variation range of Ising interface energy is Δ*G*_max_ = 3 RT. (b) The capacity of three pathways in the comprehensive ensemble model when the interface interactions are Ising-like. The variation range (Δ*G*_max_) for free-energy parameters is 8 kcal/mol.

The choice of the form of interaction is also important and may affect the results. The pristine EAM model utilizes a simplifying assumption that the state I does not involve any interface interaction. On the other hand, other schemes are possible. For example, LeVine and Weinstein have proposed an Allosteric Ising Model (AIM) where an Ising-like interaction was adopted to greatly facilitate analyses [57]. Here, we incorporate AIM to demonstrate the influence of the interaction scheme. In the spirit of AIM, the allosteric ligand A can be regarded as regular component (in a role similar to the protein domains) of the system and adopts two states: on and off. Therefore, if we incorporate the essence of AIM into the MWC model, the system has four possible states: (on/off)(R)(R), (on/off)(T)(T), while the incorporation into the EAM results in six states: (on/off)(R)(R/I), (off)(I)(R/I). With an Ising-like interaction among components, *α* is given as

$$\begin{array}{c} \alpha_{\mathrm{MWC}}=e^{-\frac{4E_{AR}}{RT}}\\ \alpha_{\mathrm{EAM}}=e^{-\frac{2E_{RR}}{k_{B}T}}\frac{e^{\frac{E_{AR}+E_{RR}}{RT}}+e^{-\frac{E_{AR}+E_{RR}}{RT}}}{e^{\frac{E_{AR}-E_{RR}}{RT}}+e^{-\frac{E_{AR}-E_{RR}}{RT}}} \end{array}$$ (17)

where *E*_*AR*_ and *E*_*RR*_ represent the Ising interaction parameters of the ligand-domain and domain-domain interface. The distributions are shown in Fig 10(A) when *E*_*AR*_ and *E*_*RR*_ randomly vary in a range of [Δ*G*_max_, −Δ*G*_max_] with Δ*G*_max_ = 3 *RT*. The MWC model has a much higher probability to achieve strong allostery than the EAM model even if the Ising-like interaction of AIM was incorporated. For the comprehensive ensemble model with three states for each domain, a Potts model (the generalized of the Ising model to > 2 states per component) can be introduced to describe the domain-domain interaction [the ligand has only two states and it is assumed to be unaffected by the Potts model here, i.e., Eq (3) is reserved], and the results are shown in Fig 10(B). The peaks near the positive allostery limit *CR*_max_ are higher than those before introducing Ising-like interaction (comparing Fig 10(B) and Fig 6(B)), indicating that an Ising-like interaction will enhance the probability of strong allostery. What is unaffected is the trend that *P*_MWC_(*CR*) is much larger than *P*_EAM_(*CR*) for strong allosteric effects. In other words, the importance of the MWC pathway over the EAM pathway is not influenced by the interaction scheme in this case.

## Discussion

### Possible reasons for the prevalence of IDPs/IDRs in allosteric regulation

IDPs/IDRs appear in much higher amounts in regulatory proteins [21,25]. They are also widely involved in allosteric processes [38–43,45,47], although it is unclear whether they are involved more widely than order proteins do. A possible explain for the prevalence of IDPs/IDRs in allosteric regulation was provided by the EAM model which suggested that intrinsic disorder can maximize the ability to allosteric coupling [12]. However, our comprehensive ensemble model reveals that the order-disorder transition (EAM mechanism) is actually less competitive than the order-order transition (MWC mechanism) in affording allosteric effects, especially the strong allostery. It shows that the reasons for the prevalence of IDPs/IDRs in allosteric regulation are more complicated than previously thought in EAM. Our work does not give a complete answer for it, but we provide some discussion and comments here.

Firstly, in our study we assumed that the free energy parameters of conformation change and domain-domain interaction (Δ*G*_R1_, Δ*G*_R2_, Δ*G*_RT1_, Δ*G*_RT2_, Δ*g*_int,R_, Δ*g*_int,T_) vary randomly with an equal probability density between [–Δ*G*_max_, +Δ*G*_max_]. In real proteins it does not have to be like this. The difficulty (probability) to modify order-order and order-disorder transitions is likely different. Specifically, to tune the protein stability difference between two similar order structures (R and T in the MWC model) via mutation would be more difficult than to tune the stability difference between order and disordered structures (R and I in the EAM model), because in the latter case this can be accomplished via breaking or strengthening a residue-residue interaction that is present in ordered structure but absent in disordered structure. Therefore, a possible reason for the prevalence of IDPs/IDRs in allosteric regulation is their convenience in modifying state stability.

Secondly, IDPs/IDRs possess various advantages over ordered proteins [26,62], such as saving genome resources via multi-binding pattern or creating large binding surface, overcoming steric effect in binding, accelerating binding speed, achieving high specificity with low affinity, and facilitating posttranslational modifications. The prevalence of IDPs/IDRs in allosteric regulation is determined by all their advantage, but not only by their capacity in endowing allostery. Work combining experimental data and bioinformatics analyses would be helpful to compare ordered and disordered proteins’ importance in allosteric regulation.

Thirdly, due to the lack of systematic survey, at present it is still unclear whether IDPs/IDRs are more or less prevalent in allostery than ordered proteins. They might play a complementary role to expand the proteome of allostery even if IDPs/IDRs do not exceed ordered proteins.

Lastly, allosteric effects with maximal *CR* may be not the pursuing goal. Allostery with different strength would have different applications. For example, allosteric effect that are not too strong is beneficial in ensuring safer dosing [63].

### Conclusions

In this work, we proposed a comprehensive ensemble model to study the role of order-order and order-disorder transitions in allosteric effect. An analytic equation for the maximal allosteric coupling response (*CR*) was derived, which shows that too stable or too unstable state is unfavorable to achieve allostery. By sampling the parameter space, it was revealed that the order-order transition (MWC) mechanism has a higher possibility in allostery than the order-disorder transition (EAM) mechanism, and the remaining mixed (Others) mechanism involving both order-order and order-disorder transitions also has a high possibility close to the MWC one. In addition, two-state transition is the primary mechanism when allostery is strong although there are seven states in the model. The work not only provided insight in understand the prevalence of IDPs/IDRs in allosteric regulation, but is also helpful for rational design of allosteric drugs.

## Supporting information

S1 Text *CR* formula of subsystems under A-T binding mode and the general proof on the maximal *CR* in the comprehensive ensemble model. (PDF)
