# Supplementary material for: A comprehensive ensemble model for comparing the allosteric effect of ordered and disordered proteins
Source: PLoS Comput Biol. 2018 Dec 3;14(12):e1006393. doi: 10.1371/journal.pcbi.1006393 (PMC6292653; doi:10.1371/journal.pcbi.1006393)
Supplement: S1 Text — (PDF) [file pcbi.1006393.s001.pdf]

Supporting Information

A comprehensive ensemble model for comparing the allosteric effect of ordered and disordered proteins

Luhao Zhang[a,b], Maodong Li[c] and Zhirong Liu[a,c,d]*

[a] College of Chemistry and Molecular Engineering, Peking University, Beijing 100871, China

[b] Department of Chemistry, Princeton University, Princeton, NJ, 08544, USA

[c] Center for Quantitative Biology, Peking University, Beijing 100871, China

[d] State Key Laboratory for Structural Chemistry of Unstable and Stable Species, Beijing National Laboratory for Molecular Sciences (BNLMS), Peking University, Beijing 100871, China

Allosteric coupling response (*CR*) of subsystems and the contribution weight under the A-T binding mode

Under the A-T binding mode, the allosteric coupling response (*CR*) of three subsystems is calculated with

$$\begin{cases} CR_{\text{MWC}} = \dfrac{P^{(\text{MWC})}_{\text{RR,[A]}} - P^{(\text{MWC})}_{\text{RR,[A]=0}}}{-\Delta g_{\text{Lig,A}} \big/ RT} \\ CR_{\text{EAM}} = \dfrac{P^{(\text{EAM})}_{\text{RR+IR,[A]}} - P^{(\text{EAM})}_{\text{RR+IR,[A]=0}}}{-\Delta g_{\text{Lig,A}} \big/ RT} \\ CR_{\text{Others}} = \dfrac{P^{(\text{Others})}_{\text{RR,[A]}} - P^{(\text{Others})}_{\text{RR,[A]=0}}}{-\Delta g_{\text{Lig,A}} \big/ RT} \end{cases}, \tag{S1}$$

where the superscript "(MWC)", "(EAM)" and "(Others)" indicates that the related probabilities of states are normalized within the MWC, EAM and Others subsystems, respectively. The contribution of pathways to the total allostery of the comprehensive system is defined as:

$$\begin{cases} Weight_{\text{MWC}} = \min(P_{\text{RR+TT, [A]=0}}, P_{\text{RR+TT+ATT, [A]}}) \times CR_{\text{MWC}} / CR_{\text{tot}} \\ Weight_{\text{EAM}} = \min(P_{\text{RR+RI+IR+II, [A]=0}}, P_{\text{RR+RI+IR+II, [A]}}) \times CR_{\text{EAM}} / CR_{\text{tot}} \\ Weight_{\text{Others}} = \min(P_{\text{RR+TI+IT, [A]=0}}, P_{\text{RR+TI+ATI+IT, [A]}}) \times CR_{\text{Others}} / CR_{\text{tot}} \end{cases}. \tag{S2}$$

Limits for the maximal *CR* in the comprehensive ensemble model

Here we discuss the limit of *CR* in the comprehensive ensemble model and prove the general validity of Eq. (8).

In general, we can classify all states into four groups: RR, OR, RO, and OO. "O" on the left side represents all states of the left domain that could not bind ligand A, i.e., it represents T and I under the A-R binding mode. "O" on the right side represents all states of the right domain that could not bind substrate B. Then we have

$$CR=\left[\frac{P_{RR}e^{-\Delta g_{\mathrm{Lig,A}}/RT}+P_{OR}}{\left(P_{RR}+P_{RO}\right)e^{-\Delta g_{\mathrm{Lig,A}}/RT}+P_{OR}+P_{OO}}-\frac{P_{RR}+P_{OR}}{P_{RR}+P_{RO}+P_{OR}+P_{OO}}\right]\cdot\frac{1}{-\Delta g_{\mathrm{Lig,A}}/RT}\,. \tag{S3}$$

Using (conducting) the normalization condition

$$P_{RR}+P_{RO}+P_{OR}+P_{OO}=1, \tag{S4}$$

P_{OO} can be eliminated from Eq. (S3) to give

$$\begin{aligned}CR&=\left[\frac{P_{RR}e^{-\Delta g_{\mathrm{Lig,A}}/RT}+P_{OR}}{\left(P_{RR}+P_{RO}\right)e^{-\Delta g_{\mathrm{Lig,A}}/RT}+1-\left(P_{RR}+P_{RO}\right)}-\left(P_{RR}+P_{OR}\right)\right]\cdot\frac{1}{-\Delta g_{\mathrm{Lig,A}}/RT}\\&=\left[\frac{P_{RR}e^{-\Delta g_{\mathrm{Lig,A}}/RT}+P_{OR}}{1+\left(P_{RR}+P_{RO}\right)\left(e^{-\Delta g_{\mathrm{Lig,A}}/RT}-1\right)}-\left(P_{RR}+P_{OR}\right)\right]\cdot\frac{1}{-\Delta g_{\mathrm{Lig,A}}/RT}\end{aligned}\,. \tag{S5}$$

The partial derivatives of *CR* with respective to P_{OR} and P_{RO} are

$$\begin{cases}\dfrac{\partial CR}{\partial P_{OR}}=\left[\dfrac{1}{1+\left(P_{RR}+P_{RO}\right)\left(e^{-\Delta g_{\mathrm{Lig,A}}/RT}-1\right)}-1\right]\cdot\dfrac{1}{-\Delta g_{\mathrm{Lig,A}}/RT}\le 0\\[2ex]\dfrac{\partial CR}{\partial P_{RO}}=\left[-\dfrac{\left(P_{RR}e^{-\Delta g_{\mathrm{Lig,A}}/RT}+P_{OR}\right)\left(e^{-\Delta g_{\mathrm{Lig,A}}/RT}-1\right)}{\left[1+\left(P_{RR}+P_{RO}\right)\left(e^{-\Delta g_{\mathrm{Lig,A}}/RT}-1\right)\right]^2}\right]\cdot\dfrac{1}{-\Delta g_{\mathrm{Lig,A}}/RT}\le 0\end{cases} \tag{S6}$$

for $\Delta g_{\mathrm{Lig,A}} < 0$ and $0 \le P_{OR},P_{RO},P_{RR} \le 1$. Two equations in (S6) are equal to 0 only for $P_{RR} = P_{RO} = 0$ and $P_{RR} = P_{OR} = 0$, respectively. Therefore, *CR* increases with decreasing P_{OR} and P_{RO}, and a necessary condition for maximal *CR* is that P_{OR} and P_{RO} reach their minimal values, i.e., $P_{OR} = P_{RO} = 0$. With such a condition, the problem of maximizing Eq. (S5) degenerates to that of maximizing Eq. (7), and it is straightforward to obtain the maximal *CR* as Eq. (8) under optimized P_{RR} as Eq. (9).

By extending the analyses, it is clear that the maximal *CR* of the EAM model or the comprehensive ensemble model would be smaller than the maximal *CR* of the MWC model if $P_{OR} = P_{RO} = 0$ is not allowed, e.g., when the stability free-energy parameters (ΔG_{R1}, ΔG_{R2}, ΔG_{RT1}, ΔG_{RT2}, $\Delta g_{\mathrm{int,R}}$, $\Delta g_{\mathrm{int,T}}$) vary randomly between [–8, +8] kcal/mol. The introduction of more additional conformations is unfavorable for achieving maximal *CR*.

To get the minimization (lower limit) of *CR*, we use the following substitution

$$\begin{cases} \tilde{P}_{RR} = P_{RR} e^{-\Delta g_{\mathrm{Lig,A}}/RT} \\ \tilde{P}_{RO} = P_{RO} e^{-\Delta g_{\mathrm{Lig,A}}/RT} \\ \tilde{P}_{OR} = P_{OR} \\ \tilde{P}_{OO} = P_{OO} \end{cases}, \tag{S7}$$

and Eq. (S3) becomes

$$\begin{aligned} CR &= \left[\frac{\tilde{P}_{RR} + \tilde{P}_{OR}}{\tilde{P}_{RR} + \tilde{P}_{RO} + \tilde{P}_{OR} + \tilde{P}_{OO}} - \frac{\tilde{P}_{RR} e^{-\Delta g_{\mathrm{Lig,A}}/RT} + \tilde{P}_{OR}}{\left(\tilde{P}_{RR} + \tilde{P}_{RO}\right) e^{-\Delta g_{\mathrm{Lig,A}}/RT} + \tilde{P}_{OR} + \tilde{P}_{OO}} \right] \cdot \frac{1}{-\Delta g_{\mathrm{Lig,A}}/RT} \\ &= \left[\frac{\tilde{P}_{RR} + \tilde{P}_{OR}}{\tilde{P}_{RR} + \tilde{P}_{RO} + \tilde{P}_{OR} + \tilde{P}_{OO}} - \frac{\tilde{P}_{RR} + \tilde{P}_{OR} e^{-\Delta g_{\mathrm{Lig,A}}/RT}}{\tilde{P}_{RR} + \tilde{P}_{RO} + \left(\tilde{P}_{OR} + \tilde{P}_{OO}\right) e^{-\Delta g_{\mathrm{Lig,A}}/RT}} \right] \cdot \frac{1}{-\Delta g_{\mathrm{Lig,A}}/RT} \end{aligned}. \tag{S8}$$

With the correspondence of

$$\begin{cases} \tilde{P}_{RR} \leftrightarrow P_{OR} \\ \tilde{P}_{OR} \leftrightarrow P_{RR} \\ \tilde{P}_{RO} \leftrightarrow P_{OO} \\ \tilde{P}_{OO} \leftrightarrow P_{RO} \end{cases}, \tag{S9}$$

the minimization of Eq. (S8) is equivalent to the maximization of Eq. (S3), which is a nice symmetry. Therefore, we achieve

$$CR_{\min} = -CR_{\max} = -\frac{\left(e^{-\Delta g_{\mathrm{Lig,A}}/2RT} - 1\right)^2}{\left(-\Delta g_{\mathrm{Lig,A}}/RT\right)\left(e^{-\Delta g_{\mathrm{Lig,A}}/RT} - 1\right)}. \tag{S10}$$
